# Exploring the relationship between medical student basic psychological need satisfaction, resilience, and well-being: a quantitative study

**DOI:** 10.1186/s12909-019-1847-9

**Published:** 2019-11-05

**Authors:** Adam Neufeld, Greg Malin

**Affiliations:** 1 College of Medicine, University of Saskatchewan, 107 Wiggins Road, Saskatoon, SK S7N 5E5 Canada; 2 Department of Academic Family Medicine, University of Saskatchewan, Saskatoon, SK S7N 5E5 Canada

**Keywords:** Medical student, Basic psychological need satisfaction, Well-being, Resilience, Self-determination theory

## Abstract

**Background:**

There is increasing acknowledgment that medical training is stressful for students and can impact their well-being. An important aspect of this is self-determination and basic psychological need satisfaction. A better understanding of how medical student perceptions of the learning environment impacts their basic psychological needs for motivation, resilience, and well-being may help to create learning environments that support the needs of medical students and help them become better healthier physicians. We aim to add to the literature on this topic by examining this relationship through the lens of Self-Determination Theory.

**Methods:**

A total of 400 students from all 4 years of the medical program at our institution were invited to complete an anonymous online survey, measuring basic need satisfaction/frustration (autonomy, competence, relatedness) within the learning environment, resilience, and psychological well-being. We used analysis of variance to assess the effect of gender, age, and year on all variables, with *t*-tests to compare subgroups. Structural equation modelling was performed to test a hypothesized model in which support of medical students’ basic needs would positively relate to their resilience and well-being.

**Results:**

The response rate was 183/400 (46%). After data cleaning, 160 remained: 67 males (42%) and 93 females (58%). There were 67 first years (42%), 35 second years (22%), 30 third years (19%), and 28 fourth years (18%). The sample mean age was 25.8 years (SD = 4.1). A well-fitting model was confirmed to fit the data, χ^2^ = 3.15, df = 3, *p* = 0.369, RMSEA = 0.018, SRMR = 0.022, CFI = 0.999. Autonomy and relatedness satisfaction exerted direct effects on well-being. Competence satisfaction did so indirectly, through its direct effect on resilience. Female medical students had lower resilience scores compared to their male peers.

**Conclusions:**

When medical students perceived their learning environment as supportive to their basic needs, it was associated with an increase in their psychological well-being. Satisfaction of competence, but not autonomy or relatedness, predicted an increase in their resilience. Fostering medical students’ basic needs for motivation, especially competence, is recommended to support their resilience and well-being. Further research is required to generalize these results further.

## Background

Medical training can be stressful for students and negatively affect their ability to be resilient and psychologically well [1]. Although many programs are addressing student distress and well-being by teaching students how to be resilient and engage in wellness activities, little attention has been paid to the role of students’ basic psychological needs for motivation and how their perceptions of support or hindrance of these needs within the learning environment can impact their resilience and well-being. A better understanding of this phenomenon could help undergraduate programs and teachers share the responsibility for ensuring our learners maintain their wellness throughout their medical education, as well as avoid inadvertently adding to an already stressful and challenging program. We aim to add to the literature on this important topic by using the lens of Self-Determination Theory (SDT) to explore this relationship among undergraduate medical students, accounting for demographic effects on our variables.

Psychological well-being is concerned with perceived thriving, overcoming adversity, pursuing meaningful goals, maturing and developing as an individual, and building quality relationships with others [2]. Its study is highly relevant in medical education, with respect to student wellness. For our research purposes, well-being was operationalized using Ryff’s eudaimonic model of psychological well-being, which encompasses multidimensional aspects of well-being, such as personal growth, purpose in life, self-acceptance, environmental mastery, autonomy, and personal relations with others [2–4]. As well-being entails overcoming challenges and maintaining healthy coping strategies in response to stress, it relates closely to an individual’s resilience [5, 6].

Resilience, defined as the ability to withstand hardship and to rebound from a stressful experience, is known to promote beneficial adaptation to difficult circumstances and play a protective role from stressful adversity, thereby facilitating well-being [7]. It relates to an individual’s capacity for maintenance, recovery, or improvement in well-being in the face of life’s challenges [3]. Hence, resilience can be both a process and an outcome, with the ability to moderate psychosocial outcomes, like well-being [8]. This relationship between stressful environments and resilience in the face of prolonged hardship [9] is considered important in the present study, in terms of medical school curriculum and student psychological well-being.

Motivation is known to be a highly important fuel for a person’s persistence, creativity, and well-being, and is thus highly relevant to medical students [10]. According to SDT, human beings universally require support of three basic psychological needs for optimal motivation and well-being: autonomy (e.g. acting with a sense of volition), competence (e.g. feelings of being able to master one’s environment), and relatedness (e.g. feeling connected and related to by others). Therefore, within a given social context, satisfaction or frustration of the three basic needs is pivotal to bolstering or hindering one’s self-determination, ability to thrive, and psychological wellness [11]. Accordingly, it is well established from studies in the SDT literature, that the learning environment (curricular structures and teacher interactions that students are exposed to) in education can affect both the type and degree of motivation that students adopt, as well as their persistence and mental health [12, 13]. Although the broader motivation literature would tell us that the learning environment plays a key role in student motivation and well-being [12, 14, 15], it has scarcely been explored in the context of undergraduate medical school. We therefore consider SDT a well-fitting theoretical framework for the present study.

In a review of the SDT and medical education literature, we found studies pertaining to student study effort and academic performance [13, 16], motivation to pursue certain careers in medicine [17], and other reports highlighting the general importance of autonomy-supportive teaching in medicine [12] and how to incorporate SDT into medical education [18]. However, to our knowledge, the present investigation is the first to explore and quantify the predictive relationship between students’ perceptions of the learning environment as need-supportive or need-thwarting to their basic psychological needs for motivation (“basic needs”), their resilience, and their psychological well-being. Additionally, as numerous studies regarding medical student resilience and well-being have reported demographic differences based on gender [19, 20] and year of study in medical school [1, 21], we too sought to account for these variables in the present study.

This study may help uncover important areas for curricula to address that pertain to the motivational-psychological needs of medical students and their wellness. Findings from this work may further contribute to improving medical student resilience and well-being by helping educators avoid unknowingly contributing to medical student stress through the learning environments they create, as well as helping to implement effective student supports. Ultimately, our goal is to help facilitate student self-determination and well-being, and to help medical students become better healthier physicians and care providers [22, 23].

### Current study

We designed our statistical analyses to test the following research questions and model:

- Does satisfaction of students’ three basic needs for motivation (autonomy, competence, relatedness) predict improved resilience and, in turn, psychological well-being?
- Given medical students tend to carry a substantial amount of anxiety around academic performance and self-esteem in medical school [24, 25], could competence satisfaction be a key factor in predicting resilience and well-being?
- Are there demographic differences (i.e. gender, age, year) among medical students in their basic need satisfaction/frustration and resilience?

Our hypotheses were:

- Basic need satisfaction (competence, autonomy, relatedness) would significantly predict improved resilience and psychological well-being, and the opposite would be true when the needs of autonomy, competence, and relatedness were perceived as frustrated [15, 22, 26–28].
- No gender differences would exist in basic need satisfaction/frustration [26].
- Male medical students would score higher in resilience compared to females [19, 20, 25].

## Methods

### Participants

A total of 400 students from all 4 years of the medical program at the University of Saskatchewan were invited to complete an anonymous internet-based survey, which asked questions related to basic psychological need satisfaction/frustration, resilience, and psychological well-being. The survey was open for 8 weeks, at the end of the academic year. Students were sent two reminders to complete the survey.

### Ethical approval

This research received ethical approval from the University of Saskatchewan Research Ethics Board, prior to carrying out the study. Written informed consent was obtained from all participants prior to study participation.

### Measures

The internet-based survey distributed contained demographic questions (i.e. year of study, age, and gender), as well as three previously validated scales:

#### Basic psychological need satisfaction and frustration scale (BPNSF)

The BPNSF scale was used to measure need satisfaction/frustration during medical school. This 24-item scale assesses the degree of perceived satisfaction or frustration of the three needs of competence, relatedness, and autonomy. In terms of measurement, it is the individual’s perception of the degree to which the environment supports or hinders their basic needs, and not necessarily the actual level that their basic needs are fulfilled. The BPNSF scale was used in the current study as previous research has found that satisfaction/frustration of these basic needs is predictive of well-being [26, 27]. It has demonstrated good internal consistency and construct validity [26].

#### Connor Davidson resilience scale (CD-RISC)

The CD-RISC scale was used to measure resilience. The 10-item scale has demonstrated strong reliability [7, 29, 30]. It includes questions that deal with overcoming adversity, persistence in the face of challenges, and adapting to difficult social circumstances. We chose this scale because it reflects the challenges that students in medical school face on a regular basis, and because resilience is a known predictor of well-being [5, 6, 31].

#### Psychological well-being scale (PWB)

Ryff’s PWB inventory is a 42-item measure of well-being. It is comprised of six factors: environmental mastery, purpose in life, autonomy, positive relations, personal growth, and self-acceptance, which have all demonstrated good internal consistency and reliability [4]. Average ratings across the six factors were combined and averaged into an overall psychological well-being measure, as in other studies [32]. The PWB scale was chosen for the purpose of this study as it captures themes relevant to medical school (i.e. perceived thriving and overcoming adversity, pursuing meaningful goals, maturing and developing as an individual, and building quality relationships with others) [2–4]. It has shown good internal consistency and construct validity [9].

### Statistical analyses

The software program SPSS version 24.0 was used to carry out our basic analyses. Data cleaning was conducted, which included detection and removal of invalid or missing data. After checking for normal distribution of our data, we assessed our variables for correlation, using variance inflation factor (VIF) as a test of multicollinearity (acceptable VIF < 5.0). Reliability tests were carried out for all variables, using Cronbach’s alpha coefficients as a measure of internal consistency (ideal α > 0.70). One-way Analysis of Variance (ANOVA) was planned to assess whether age, gender, and year of study affected the need satisfaction/frustration and resilience variables. To compare scores between subgroups showing significant effects, Levene’s test of homogeneity of variance was performed, followed by unpaired *t*-tests, using Bonferroni’s *p*-value correction to adjust the family-wise error rate appropriately. Cohen’s d effect sizes were included to measure the magnitude of the mean differences between groups, where *d* = 0.2 is considered “small”, *d* = 0.5 is “medium”, and *d* = 0.8 is “large” [33].

Path analysis (structural equation modelling without latent variables) was carried out using R version 3.3.3 to compare the hypothesized and tested model, accounting for basic psychological need satisfaction/frustration, resilience, and psychological well-being. Bootstrapping procedures were used to test the significance of the structural equation model (SEM) and parameter estimates, generating 500 bootstrapping samples from the original data (*n* = 160) by random sampling, with a 95% confidence interval for the indirect and direct effects. The indices (with cutoff criteria in parentheses) used for estimating goodness of fit for the model were Chi square goodness of fit (χ^2^), Comparison of Fit Index (CFI close to 1), Root Mean Square Error of Approximation (RMSEA < 0.08), Standardized Root Mean Square Residual (SRMR < 0.08), and Chi square *p*-value (> 0.05) [34, 35].

This study was part of a larger study at our institution addressing medical student psychological well-being and each of the six factors that make up Ryff’s PWB scale. Therefore, the comparisons between subgroups (e.g. age, gender, year) in the present study pertained primarily to basic psychological needs and resilience. As described in Methods, the six psychological well-being factors were grouped together for an overall measure of well-being, and are hence reported in that context [32].

## Results

### Demographics

The response rate of the medical students was 183/400 (46%). After data cleaning (see Methods), there were 160 responses remaining—67 males (42%) and 93 females (58%). By year, there were 67 first years (42%), 35 second years (22%), 30 third years (19%), and 28 fourth years (18%). The sample mean age was 25.8 years (SD = 4.1).

### Comparisons

One-way ANOVA was carried out to explore whether gender, age, and year affected the resilience (R) and basic need variables; satisfaction of autonomy (AS), competence (CS), and relatedness (RS), and frustration of autonomy (AF), competence (CF), and relatedness (RF). Levene’s test indicated equal variances between groups based on year, gender, and age, for all three basic need satisfaction (AS, CS, RS) and frustration variables (AF, CF, RF), and for R (all *p*’s > 0.05). There were no significant effects of gender, age, or year on the need satisfaction/frustration variables (all *p*’s > 0.05). There was a marginally significant effect of gender on R, *F* (1,158) = 3.90, *p* = .050. A post hoc unpaired *t*-test revealed that male medical students (M = 30.6, SD = 5.2) scored modestly higher in R than the female medical students (M = 28.8, SD = 5.6), *t* (158) = 1.98, *p* = 0.050*, d = 0*.32. There was no significant effect of year or age on R (all *p*’s > 0.05).

### Preliminary analyses

**Fig. 1:**
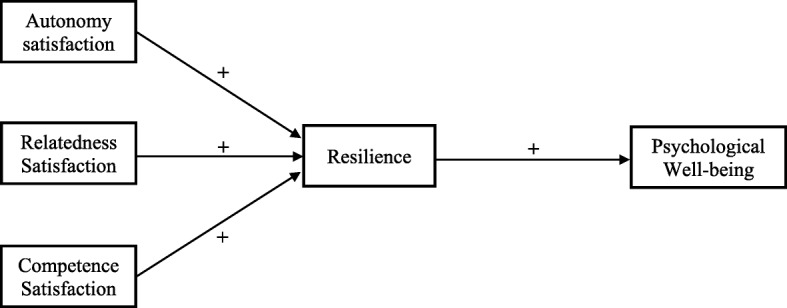
SEM depicting hypothesized relationship between satisfaction of autonomy, competence, and relatedness, resilience, and psychological well-being. * indicates statistically significant at *p* < 0.001 level. Values for each arrow indicate the standardized prediction coefficients

Listed in Table 1 are the reliability and correlation coefficients for the variables of interest. AS, CS, and RS each positively correlated with psychological well-being (PWB) and resilience (R). Conversely, AF, CF, and RF each negatively correlated with PWB and R. All need satisfaction variables negatively correlated with the need frustration variables. The independent autonomy variables from the BPNSF and PWB scales had some correlation but not enough to be concerned about in terms of accurate regression coefficient estimation in the SEM (VIF = 1.6). The demographic variables (age, gender, and year) did not correlate statistically with any other variables. These results formed the basis for testing the model proposed (see Fig. 1).

**Table 1:** Reliabilities and correlations between all variables (*n* = 160)

	α	Age	Gen	Year	AS	CS	RS	AF	CF	RF	R
Gen		−0.07									
Year		0.16	0.09								
AS	0.77	0.06	−0.01	−0.04							
CS	0.87	0.00	0.00	0.03	0.55*						
RS	0.86	−0.07	− 0.07	0.03	0.52*	0.54*					
AF	0.80	−0.02	− 0.02	0.10	−0.68*	− 0.53*	− 0.52*				
CF	0.86	0.02	0.02	0.09	−0.44*	− 0.77*	− 0.53*	0.53*			
RF	0.83	−0.07	− 0.07	− 0.12	−0.46*	− 0.52*	− 0.71*	0.54*	0.61*		
R	0.87	0.06	0.06	−0.05	0.40*	0.62*	0.37*	−0.36*	−0.58*	− 0.44*	
PWB	0.93	0.07	0.01	−0.03	0.65*	0.68*	0.74*	−0.66*	−0.71*	− 0.72*	0.60*

*Gen* Gender (0 = male, 1 = female), *Year* (1 to 4), *α* Cronbach’s alpha, *AS* autonomy satisfaction, *CS* competence satisfaction, *RS* relatedness satisfaction, *AF* autonomy frustration, *CF* competence frustration, *RF* relatedness frustration, *R* resilience, *PWB* psychological well-being

* indicates statistically significant at *p* < 0.01 level

### Primary analyses

**Fig. 2:**
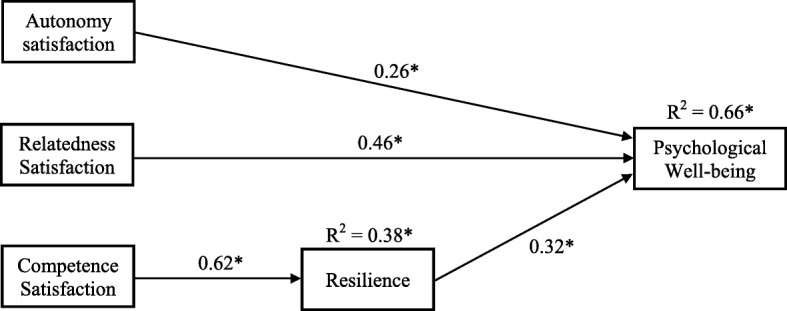
Reduced SEM depicting effects of autonomy, competence, and relatedness satisfaction on resilience and psychological well-being. * indicates statistically significant at *p* < 0.001 level. Values for each arrow indicate the standardized prediction coefficients

All 500 requested bootstrap draws were successful and converged normally after 26 iterations. Based on the model characteristics, there was a satisfactory fit to the data: χ^2^ = 3.15, df = 3, *p* = 0.369 (> 0.05), therefore non-significant (Chi square goodness of fit). The model fit parameters were CFI = 0.999 (close to 1), RMSEA = 0.018 (< 0.08), and SRMR = 0.022 (< 0.08), which is a good fit. Figure 2 shows the reduced SEM with significant effects of basic need satisfaction (autonomy, competence, relatedness) on resilience and psychological well-being. This excluded the demographic subgroups, as well as the basic need frustration variables, which were not a good fit.

As seen in Fig. 2, it was found that autonomy satisfaction (β = 0.26, *p* < 0.001) and relatedness satisfaction (β = 0.46, *p* < 0.001) exerted direct effects on psychological well-being, while competence satisfaction did so indirectly (β = 0.32, *p* < 0.001) through its direct effect on resilience (β = 0.62, *p* < 0.001). Competence satisfaction accounted for 38% of the explained variance in resilience. Put together in the model, satisfaction of autonomy, competence, and relatedness, and resilience, accounted for 66% of the variance in psychological well-being.

## Discussion

Our results demonstrate that a significant degree of medical students’ psychological well-being can come through satisfaction of their basic psychological needs for motivation (autonomy, competence, and relatedness). Further, our findings support the notion that resilience is a key factor in relation to well-being, whose association with well-being has been well documented in the literature [2–5, 19, 36, 37]. In the present model, autonomy and relatedness satisfaction had a direct effect on psychological well-being, whereas competence satisfaction had an indirect effect on well-being, through its direct effect on resilience. These results lead us to consider that medical students’ feelings of competence satisfaction (“Can I really do this?”), perhaps to a greater extent than the other two basic psychological needs, are integral to their resilience and ability to persevere in medical school.

Competence satisfaction is known to be an important factor in predicting self-determination and well-being [12, 16, 38], as it relates closely with self-esteem—a major source of stress for medical students described in the literature [25]. Given medical students must endure many challenges throughout their training, finding ways to support medical student perceived competence is important, and it likely represents a valuable avenue for supporting their resilience and well-being. Further, we propose that if we as teachers support learners’ three basic psychological needs, then there may be less of a need for students to enact resilience strategies, because they have less of a need to bounce back from or persist through difficulties beyond those inherent to the medical profession. Kusurkar et al. [18] provide a more detailed review of ways to support the three basic needs for optimal motivation, derived from SDT. Following these suggestions may subsequently serve to help educators and teachers support student resilience.

In our study, we explored whether there were any demographic differences in student resilience, as it relates to well-being. Male medical students in our sample scored modestly higher in levels of resilience compared to the female medical students. While similar findings have been reported in several other reports in the medical education literature [19, 20, 39], other studies have reported no impact of gender on resilience [40]. Why this might matter is that, despite equal performance in medical school compared to males, female medical students tend to report decreased levels of self-confidence, particularly over issues related to their feelings of competence [24, 25, 41–43], which we have shown can impact resilience. Although the effect of gender on basic need satisfaction/frustration was not found to be statistically significant, we nonetheless recommend that further research evaluate this link, as well as the potential impact of medical students’ perceptions of competence on their resilience and well-being [24].

Although it has been recommended that an increased emphasis on self-care be mandated in medical education [19], we would add to these suggestions and recommend that increased attention be paid to the learning environment that medical students must navigate and how medical school programming might best support learner psychological needs for motivation. This could be achieved by taking steps to increase faculty sensitivity toward the benefits and harms of need-supportive and need-thwarting learning environments in medical school [12], especially competence [18], and through building resilience and confidence boosting interventions into undergraduate medical curriculum [44]. Put together with other studies that have shown basic need satisfaction to predict better study effort and improved academic performance in medical school [45], we highlight the potential value for it to further benefit medical students’ resilience and eudaimonic well-being.

### Limitations

There are several limitations which may serve to guide future research. One limitation was a fairly small sample size, and that the study was carried out at a single site, thereby limiting its generalizability. Although many consider a sample size of 200 to be a minimum for structural equation modelling, determining adequate sample size in SEM should not be based on commonly cited rules-of-thumb, since it is model-dependent and relies on a range of factors beyond statistical power alone [46]. The SEM in the present study had adequate statistical power, sufficient number of cases per variable, and strong factor loadings; it is thus considered to have adequate sample size. Another limitation was the disproportionately higher response rate from the students in first year compared to other years in the program. While our variables did not show any heterogeneity of variance based on year of study, there is still potential for representation bias, given the relatively low number of respondents in the more advanced years. Therefore, caution should be used at this point when interpreting these findings, as more robust hypothesis testing and replication of this study are warranted, in terms of gathering more data to validate and further generalize these results.

With respect to the variables used in the SEM, although we set out to utilize the BPNSF scale to measure students’ basic need satisfaction and frustration as they relate to resilience and well-being, we ran into difficulty with the frustration variables in the model, due to lack of a measure for ill-being [26]. Future studies using the need frustration variables can consider incorporating an additional outcome variable to operationalize ill-being (e.g. perceived stress, burnout, etc.) [47], where the need frustration variables would be predictors. Finally, both the BPNSF and PWB scales had measures of autonomy that modestly correlated, which created the potential for multicollinearity. However, based on their low variance inflation factor, it was not deemed problematic to keep both in the analysis. We acknowledge these limitations here for the benefit of future studies assessing similar constructs. Future studies may also consider similar models but at the residency level in medical education, given those years of medical training are also known to be particularly stressful for young medical doctors.

## Conclusion

Our findings pertain to a critical topic in medical education, with the potential to help assist programs in ameliorating student motivation, resilience, and well-being, and by extension, the quality of the patient care those student doctors may provide. To that end, we suggest that medical educators consider strongly the importance of creating learning environments in medical school that support and do not hinder students’ basic psychological needs for motivation and well-being. In particular, we highlight that medical students’ resilience, and efforts toward building it, likely hinge upon their feelings of support for their competence.

## Data Availability

The datasets used and/or analysed during the current study are available from the corresponding author on reasonable request.

## Abbreviations

AF: Autonomy frustration
ANOVA: Analysis of variance
AS: Autonomy satisfaction
BPNSF: Basic psychological need satisfaction and frustration scale
CFI: Comparison of fit index
CF: Competence frustration
CS: Competence satisfaction
D: Cohen’s d effect size
DF: Degrees of freedom
M: Mean
N: Sample size
PWB: Psychological well-being
R: Resilience
RF: Relatedness frustration
RMSEA: Root mean square error of approximation
RS: Relatedness satisfaction
SD: Standard deviation
SDT: Self-determination theory
SEM: Structural equation model
SRMR: Standardized root mean square residual
VIF: Variance inflation factor
χ^2^: Chi square
